# Dimensions of patient satisfaction with comprehensive abortion care in Addis Ababa, Ethiopia

**DOI:** 10.1186/s12978-016-0259-0

**Published:** 2016-12-07

**Authors:** Bekalu Mossie Chekol, Dame Abera Abdi, Tamirie Andualem Adal

**Affiliations:** 1 Ipas Ethiopia, P.O. Box 63001, Bole, Addis Ababa, Ethiopia; 2 School of Psychology, Addis Ababa University, P.O. Box 1176, 6 killo, Addis Ababa, Ethiopia

**Keywords:** Comprehensive abortion care, Satisfaction, Factor analysis, Factors, Ethiopia

## Abstract

**Background:**

Patient satisfaction is a measure of the extent to which a patient is content with the health care received from health care providers. It has been recognized as one of the most vital indicators of quality. Hence, it has been studied and measured extensively as part of service quality and as a standalone construct. In spite of this, there has been limited or no studies in Ethiopia that describe factors of abortion care contributed to women’s satisfaction. This study aimed to identifying the underlying factors that contribute to patient satisfaction with comprehensive abortion care and at exploring relationships between total satisfaction scores and socio-demographic and care-related variables in Addis Ababa, Ethiopia.

**Methods:**

At the beginning of the study in-depth interviews with 16 participants and a focus group discussion of 8 participants were conducted consecutively at the time of discharge to generate questions used to evaluate women’s satisfaction with abortion care. Following generation of the perceived indicators, expert review, pilot study, and item analysis were performed in order to produce the reduced and better 26 items used to measure abortion care satisfaction. A total sample size of 450 participants from eight health facilities completed the survey. Principal component exploratory factor analysis and confirmatory factor analysis were conducted respectively to identify and confirm the factors of abortion care contributing to women’s satisfaction. Mean satisfaction scores were compared across socio demographic and care-related variables such as age, educational level, gestational age (first trimester and second trimester), and facility type using analysis of variance.

**Results:**

Exploratory factor analysis of the 26 items indicated that satisfaction with abortion care consisted of five main components accounting for 60.48% of the variance in total satisfaction scores. Factor loadings of all items were found to be greater than 0.4. These factors are named as follows: “art of care” which means interpersonal relationships with the care-provider, “physical environment” which means the perceived quality of physical surroundings in which care is delivered, including cleanliness of facilities and equipment, “information” which means the information received related to abortion procedures, “privacy and confidentiality”, “quality of care” which refers to technical quality of the care provider. Furthermore, analysis of variance showed that overall satisfaction is found to be related to facility type, relationship status, gestational age, and procedural type.

**Conclusion:**

The findings provided support that women’s satisfaction with comprehensive abortion care has five major factors. Therefore, to improve the overall quality of comprehensive abortion care, attention should be given to the advancement of these components namely, positive interpersonal communication with care-receiver, pleasantness of physical environment, offering enough information related to the procedure, securing clients’ privacy during counseling and treatment, and technical quality of the providers.

## Plain English summary

Patient satisfaction measures the extent to which a patient is content with the health care received from health care providers. It has increasingly been recognized as one of the most vital signs of quality health care services. The purpose of this study was to identify the underlying factors that contribute to patient satisfaction with comprehensive abortion care and at exploring relationships between satisfaction and socio-demographic and care-related characteristics in Addis Ababa, Ethiopia. Factor analysis were conducted to identify and confirm factors contributing to women’s satisfaction with comprehensive abortion care. Mean satisfaction scores were compared across socio demographic and care-related variables. The findings provided support that women’s satisfaction with comprehensive abortion care has five major factors namely art of care, physical environment, information, privacy, and quality of care. Further analysis showed that satisfaction with abortion care is related to facility type, relationship status, gestational age, and procedural type. However, satisfaction with abortion services is not found to be related with age group, level of education, and diagnosis. Therefore, to improve quality of abortion care, attention should be given to positive interpersonal communication with care-receiver, pleasantness of physical environment, offering adequate information related to the procedure, securing clients’ privacy during counseling and treatment, and technical quality of the providers.

## Background

Despite the advancements in health technologies, health evidence and human rights justification for providing comprehensive abortion care (CAC), unsafe abortion remains a major public health concern. According to the World Health Organization (WHO), 22 million unsafe abortions are performed each year; nearly all of them (98%) occur in developing countries [1]. Approximately, 47, 000 deaths are due to unsafe abortion complications [1]. In addition, five million women are estimated to suffer disability because of complication due to unsafe abortion [2]. In developing countries, nearly 13% of maternal mortality is caused by unsafe abortion [2]. Ethiopia is one of the developing countries with the highest mortality where unsafe abortion account for 32% of all maternal deaths [3].

According to the WHO, almost every one of the deaths and morbidities caused by unsafe abortion could have been prevented thorough effective sexual education, family planning services, and provision of safe, legal induced abortion and treatment of abortion complications [2]. The Ethiopian Parliament in 2004 relaxed the former absolute-abortion-prohibitive code by legalizing (Article 551: 1) abortion under the following conditions: when pregnancy is as a result of rape or incest; when continuance of the pregnancy endangers the health or life of the mother or the fetus; in cases of fetal abnormalities; for women with physical or mental disabilities; for minors who are physically or psychologically unprepared to raise a child; in cases of grave and imminent danger that can be averted only through immediate pregnancy termination [4].

For abortion care to be effective, continuous service improvement strategies need to be in place as part of maintaining service quality to meet health care needs and rights of women [5]. Studies have shown that poor service quality results in low acceptability of legal abortion services that may lead women to seek care from unqualified providers or to self-induce abortions, which can result in abortion-caused morbidity and mortality [6]. Despite the expansion of safe and legal abortion services in Ethiopia, a study conducted in 2008 estimated that one in ten pregnancies would end in abortion, and 73% of these abortions assumed to be performed unsafely outside health facilities [7]. The risk of death following unsafe abortion procedures is by far higher than that of abortion carried out professionally [8]. Service quality may also influence such factors as women’s interest to return to abortion care and to practice post abortion services. Moreover, clients may share their bad experiences with friends and family and create a negative reputation for legal services in the health facilities that may lead women to look for illegal abortions [9].

To make abortion services fit the needs of women thereby avoid women dissatisfaction, a client-centered approach has emerged as a critical component of service quality improvement tactics. Health care managers thus need to take women’s opinions into account when designing service quality improvement strategies [10]. Therefore, evaluating abortion care satisfaction is a legitimate approach that can differentiate factors to be addressed for advancing service qualities [11].

So far studies done in Ethiopia have focused mainly on the cause, magnitude, and distribution of abortion services. In addition, while several studies have examined patient satisfaction with other types of healthcare services [6, 7], there is limited information available about factors contributing to women’s satisfaction with CAC in Ethiopia. This study will identify major factors that describe CAC services from the perspectives of consumers and explore differences in the mean satisfaction scores among groups of selected socio demographic and care-related variables.

## Methods

### Study participants and location

This facility based cross-sectional study utilized formative qualitative and quantitative methods. The study was conducted in Addis Ababa city administration, between the months of January to November, 2014. Health facilities were chosen based on availability of CAC service and high caseloads. The number of women accessing abortion services during the month prior to the study was used to select high caseload facilities. Based on these criteria, four public or governmental facilities (of which two were referral hospitals) and four private or non-governmental organization (NGO) led facilities were selected. While all the selected facilities offer medical abortion (MA) and manual vacuum aspiration (MVA), second-trimester abortions (Ministry of Health of Ethiopia defined this as beyond 12 weeks’ gestation by ultrasound) are limited to two referral hospitals.

For interviews and one focus group discussion, selection of participants was purposive to maximize variation and participants with rich data. Health workers assisted in recruitment of participants. Interviews with 16 clients (two from each facility) and a focus group discussion with eight clients (one from each facility) were conducted consecutively at the time of discharge. Women who participated in the focus group discussion and interviews were not included in the survey.

The sample size for quantitative survey was determined after considering varying suggestions and several guiding rules made by numerous scholars, such as recommendations that a sample size of more than 100 is adequate to run factor analysis, and the recommendation that adequate sample size can be determined using 10:1 (sample to variable) ratio [12–14]. Accordingly, the total sample size was 450. The sample at each facility was proportionate to the number of abortions performed the previous month. All women, receiving abortion services from the selected health facilities within data collection period were approached and asked if they would like to participate until the allocated number was reached. Women receiving abortion care by any method at any ages of gestation were included in the study. Women who refused to participate or were unable to respond because of abortion related complications were excluded from the study.

For the sake of factor analysis, the 400 fully completed questionnaires were divided into two using SPSS 18 (Data, Select Cases, Random Sample of Cases- 50%). The first sample used for exploratory factor analysis was 191 and the second sample used for confirmatory factor analysis was 209.

### Data collection procedure

Interviews and focus group discussion were applied in respective order to assess women’s experience on CAC. Interviews were conducted by using open-ended questions to elicit clients’ comments about the positive and negative aspects of care received. Note taking and tape recording were used. Five of the sixteen interviewees declined to be tape recorded. The reason they gave for declining was it was their first time as a result they would not be at ease. Concepts and themes captured from the interview served as the basis for the focus group discussion. Women who participated in the interviews were not included in the focus group discussion.

After identification of major concepts and themes obtained from the interview as well as literature and preparation of guiding questions, one focus group discussion of eight participants was carried out. These participants were selected with the help of clinicians on the basis of their experience and ability to give opinion clearly and thoroughly. Once their consent was secured, all of them were invited to the office of the principal investigator and discussed their experience on CAC for about 90 min.

Following reading of the notes several times, summarizing and filtering forty items were generated. Two experts who had a second degree in measurement and evaluation and working in health care management reviewed the forty items. Based on their individual and common remark, thirty five items were selected. These thirty five items were tested using 30 participants at the time of discharge and the necessary item analyses (frequencies, means, standard deviations, item-total correlations and corresponding reliability values) were made. The net effect of this item development processes resulted in a 26-item questionnaire**.**

The final questionnaire contained two parts. The first part included socio-demographic and care-related variables (such as age, marital status, educational level, facility type, diagnosis type, procedure type, and gestational age). The second part contained items related to CAC, ranging from reception to discharge. Participants were asked to respond by indicating their level of satisfaction on a four-point ‘Likert-type scale’, ranging from “strongly disagree ” (1) to “strongly agree” (4).

Ethics approval was sought and obtained from Addis Ababa University and all ethical standards for human subject research were adhered to throughout the study period. During data collection, eight health care professionals, who were not working on CAC-related services, conducted the interview. Informed consent was obtained from clients who participated in the study and their right of refusal to participate in or withdrawing from the study at any stage was maintained. Confidentiality of responses was maintained, and personal privacy and cultural norms was also respected properly.

### Statistical analysis

Data were entered in to Epi Info 3.5.1 and exported to SPSS 18.0 for statistical analysis. Data exploration was done to assess the general descriptive features of the data. After exploration, the mean, median, standard deviation, and total scores of satisfaction were computed. Item scale correlation, communality, and factor loadings were also calculated. Mean satisfaction scores were used to assess level of satisfaction among different groups of women.

To extract factors underlying women’s satisfaction with CAC three statistical techniques were used: (1) Principal component exploratory factor analysis (EFA) using varimax rotation, (2) Scree plot graph, (3) Monte Carle parallel analysis. In exploratory factor analysis, only those factors that explain an appreciable amount of variance, factors with eigenvalues greater than 1.0 were retained. Prior to extraction of factors, Kaiser-Meyer-Olkin (KMO) measure of sampling adequacy and Bartlett’s Test of Sphericity were employed to assess suitability of data for factor analysis.

Following exploratory factor analysis confirmatory factor analysis was performed using AMOS (Analysis of Moment Structure) software. Satisfaction differences between levels of the selected socio-demographic and care-related characteristics were tested using analysis of variance. Level of significance (*p*-values) less than 0.05 were considered to be statistically significant.

## Results

Of the 450 women recruited to participate 400 (88.9%) completed the survey. Women with incomplete data (15 were unable to finish the interview because of sickness and 35 declined after the interview was started) were not included in the analysis. The mean age of participants was 25.3 + 4.9 years. While 160 (40%) were from private clinics and 120 (30%) public facilities, Marie Stopes clinics contributed 120 (30%) of the respondents. Majority of the cases (89%) were first trimester (Ministry of Health of Ethiopia defined this as within 12 weeks’ gestation by ultrasound), safe induced abortion (81%) and with education ranging from no formal education (12.5%) to university/college graduate (16.2%), high school graduates constituted the highest proportion (33.5%). Nearly one half of the procedures were medical abortions and the rest were manual vacuum aspirations. The distribution of respondent characteristics and their proportion is displayed in Table 1.

**Table 1:** Demographic and care-related characteristics of study participants (*N =* 400)

Client Characteristic	*N* (%)
Age (*n =* 400)	
15–19	49 (12.25)
20–24	136 (34)
25–34	187 (46.75)
35–45	28 (7)
Diagnosis type (*n =* 400)	
Induced Abortion	325 (81.3)
Post Abortion Care	75 (18.7)
Gestational age^a^ (*n =* 400)	
First Trimester	356 (89.0)
Second Trimester	44 (11.0)
Procedure type (*n =* 400)	
Medical abortion	195 (47.9)
Manual Vacuum Aspiration	205 (52.1)
Facility type (*n =* 400)	
Public	120 (30.0)
Private	160 (40.0)
Marie Stopes (NGO)	120 (30.0)
Relationship status^b^ (*n =* 381)	
Married	190 (50.13)
Living with partner	191 (49.87)
Educational level (*n =* 400)	
No formal education	49 (12.5)
Primary school	89 (22.7)
Secondary school	135 (33.5)
Technical school	61 (15.0)
University/College Graduate	66 (16.2)

^a^First Trimester means less than or equal to 12 weeks of gestation and Second Trimester refers to greater than 12 weeks of gestation

^b^Numbers may not sum to 400 due to missing data

Although respondents used the full range of responses (one to four) to each of the 26 satisfaction items, the individual means of items were relatively high, ranging from 2.86 to 3.50 (standard deviations from 0.59 to 0.92), and majority of them (65%) were skewed toward upper tail indicating that majority of women reported higher rates of satisfaction. The overall mean of items was 3.27 and variance of the item means was 0.52. The mean score of the entire satisfaction items was 85.06 (*SD* = 9.57, median *=* 84.00), with a range between 48 and 96.

### Extraction of factors

To assess the fitness of data for factor analysis, Kaiser-Meyer-Olin (KMO) index was computed and found to be 0.79, indicating that the data was suitable for factor analysis. The Bartlets test of Spherity was also statistically significant (*X* ^2^ = 2265.66, df = 325, *P <* 0.001), showing that the inter-item correlation matrix was not an identity matrix and factor analysis was appropriate.

**Fig. 1:**
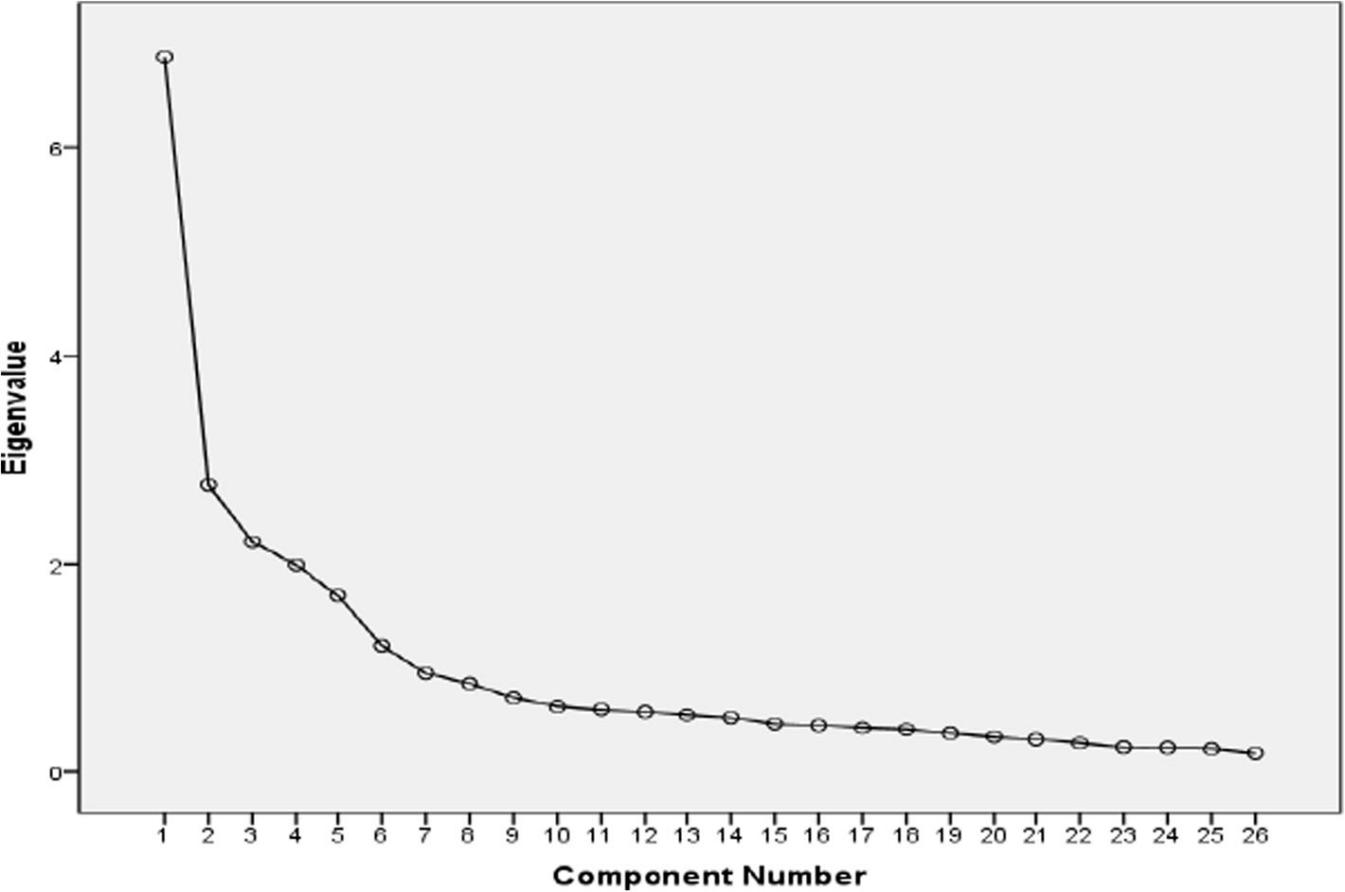
Scree Plot Graph

Using principal component factor analysis six factors with eigenvalues ranging from 1.10 to 6.46 were extracted. The total percentage of variance explained by these factors was 63.6%. Further analysis was made to determine number of factors to be retained. First, the Scree plot method showed a clear separation between factors 5 and 6, where the scree appears to begin, suggesting a five-factor solution (Fig. 1). Second, the observed eigenvalues of the six factors identified by factor analysis were compared with values derived by using Monte Carlo PCA parallel analysis method, only eigenvalues of the first five factors exceeded their counterparts, indicating that the last (6th) factor should be ignored. Thus, the exploratory stage of the study has come up with five-factor solution that explain 60.48% of the total variance. The identified factors and their corresponding items are reported in Table 2.

**Table 2:** Varimax rotated principal component matrix (*N =* 191)

	Component				
	1	2	3	4	5
4. The health care provider explained my procedure/care to me in a way that I easily understood.	.83				
2. The health care provider didn’t encourage me to talk about all my problems and concerns. (R)	.80				
3. The staff at this facility was welcoming and made me feel comfortable with my care.	.76				
13. Health care provider seemed to want get read of me as soon as possible. (R)	.68				
9. The health care provider didn’t show respect to what I have to say. (R)	.66				
8. The health care provider has asked me if I have questions and concerns about the procedure.	.58			.48	
1. The health care provider treated me in a very friendly and courteous manner.	.56				
11. Health care provider really cares about me as a person.	.49			.49	
25. I feel the atmosphere of the procedure room is good.		.88			
5. The Procedure room was not comfortable and attractive. (R)		.85			
12. Health facility is not conveniently located. (R)		.77			
19. Facilities and equipment around the procedure area are not tidy. (R)		.74			
23. The waiting room seats are uncomfortable. (R)		.70			
18. There are clear signs and direction to indicate where to go in the service areas.			.77		
6. The health care provider told me that without using a contraceptive method I could get pregnant again.			.75		
22. Staffs at the reception ease me to obtain all information I need about the service.			.74		
14. Health care provider has not given me enough information about the care so that I didn’t know what to expect. (R)			.68		
17. The health care provider didn’t tell me about follow-up care for when I get home. (R)			.66		
15. Feel enough privacy while being treated.				.81	
24. I feel comfortable that no one could observe from outside during examination and procedure had been done.				.73	
16. I suspect others could listen during counseling and/or procedure had been done. (R)				.63	
26. I did not feel free during examination and procedure since it was interrupted by others. (R)				.57	
21. The health care provider has given me medicine to help relieve pain during my procedures.					.83
20. I am not confident of the ability of the provider who treat me. (R)					.75
7. I doubt that procedure room has adequate medical instruments and equipment needed to provide complete care. (R)					.63
10. Health care providers and their staffs were available during my visit.					.60

**Fig. 2:**
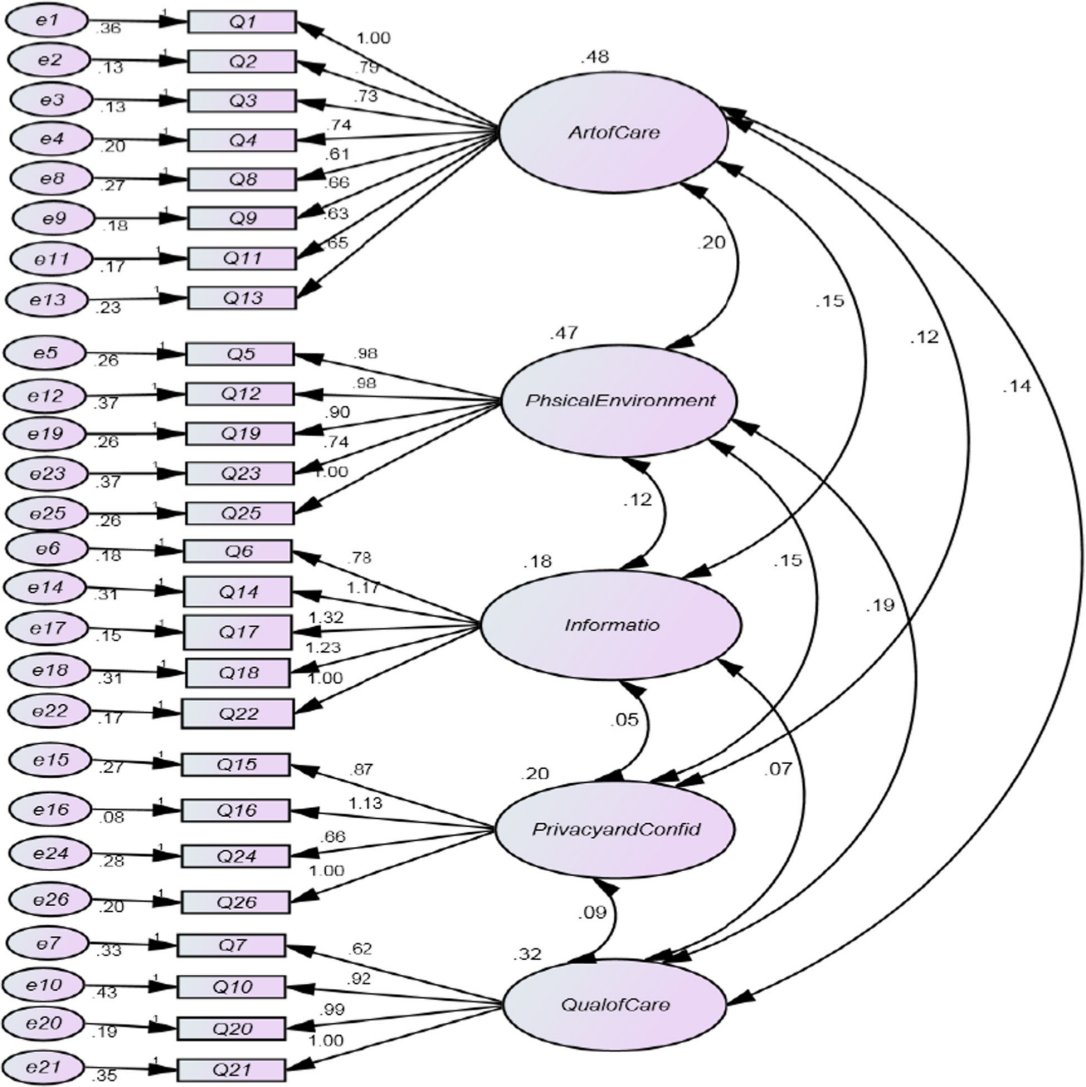
Confirmatory Factor Analysis Model

The confirmatory factor analysis as seen below (Fig. 2) showed a moderate level of fitness to the data as shown in the following indices or measures: *χ*2 (df = 286) = 855.32, *P <* .001; GFI = .771; RMR = .037; RMSEA = .097; PNFI = .65; PCFI = .71

The five factors were labeled as art of care, physical environment, information, privacy and confidentiality, and quality of care.

The art of care consisted of eight items (item 1, 2, 3, 4, 8, 9, 11, and 13) with factor loadings ranging from 0.49 to 0.83. Items in this component emphasized on the importance of interpersonal manner of the provider on women’s satisfaction with care. This factor indicated the way a provider communicates with his or her client has a great impact on satisfaction. It included statements of being welcoming, caring, and showing respect.

The physical environment consisted of five items (items 5, 12, 19, 23, and 25) with factor loadings ranging from 0.70 to 0.88. Items in this dimension focused on satisfaction with the physical surroundings in which care is delivered. Items in this factor described physical environment as general pleasantness, comfort, attractiveness, and conformableness with the procedure and waiting room, including cleanness of facilities and equipment.

The information component consisted of five items (items 6, 14, 17, 18, and 22) with factor loadings ranging from 0.66 to 0.77. Statements in this factor stated the kind of information received about care, including follow-up care and post abortion services. It also reflects the need to simplify things to women by offering the desired information related to the procedure.

The privacy and confidentiality consisted of four items (items 7, 10, 20 and 21), having factor loadings ranging from 0.60 to 0.83. Those items described how woman’s privacy was secured while she was being counseled and treated.

The quality of care comprised of four items (items 15, 16, 24 and 26) having factor loadings from 0.57 to 0.81. This dimension which also described the availability of adequate medical instruments and supplies assessed women’s perceptions on competence of service providers and their adherence to high standards of diagnosis and treatment.

### Satisfaction among different groups of women

Analysis of variance was applied to compare mean satisfaction scores across socio-demographic and care-related characteristics (Table 3). Significant differences were found between type of facility, relationship status, procedure type, and gestational age (*P <* 0.05). As such, there was no significant difference observed on satisfaction scores between age group, level of education, and type of diagnosis (*p* > 0.05).

**Table 3:** Mean satisfaction scores of study participants (*N =* 400)

Characteristic	Mean (SD)	*P* ^1^
Age (*n =* 400)		0.127
15–19	82.88 (9.86)	
20–24	84.77 (9.84)	
25–34	85.40 (8.97)	
35–45	88 (11.0)	
Diagnosis type (*n =* 400)		0.51^2^
Induced Abortion	85.21 (9.41)	
Post Abortion Care	84.40 (10.24)	
Gestational age (*n =* 400)		<0.001^3^
First Trimester	84.51 (9.22)	
Second Trimester	89.50 (11.18)	
Procedure type (*n =* 400)		0.002^4^
Medical abortion	83.53 (9.35)	
Manual Vacuum Aspiration	86.51 (9.57)	
Facility type (*n =* 400)		<0.001^5^
Public	89.61 (11.54)	
Private	83.39 (8.06)	
Marie Stopes (NGO)	82.73 (7.53)	
Relationship status (*n =* 381)		<0.01^6^
Married	86.32 (9.64)	
Living with partner	83.52 (9.21)	
Educational level (*n =* 400)		0.203^7^
No formal education	87.08 (9.44)	
Primary school	86.97 (9.00)	
Secondary school	84.51 (9.93)	
Technical school	83.48 (8.78)	
University/College Graduate	83.56 (9.91)	

^1^Probability of the given result or less

^2^Satisfaction scores of physical environment were related with diagnosis type (*P <* .001)

^3^All factors’ satisfaction scores except privacy and confidentiality were found to be related with gestational weeks (*P <* .05)

^4^Apart from physical environment and privacy and confidentiality, all factors’ satisfaction scores were related with procedure type (*P <* .05)

^5^Except physical environment all factors’ satisfaction scores were related with facility type (*P <* 0.05)

^6^Only Physical environment and privacy and confidentiality scores were not related with relationship status (*P <* .05)

^7^All factors’ satisfaction scores except quality of providers were not found to be related with educational levels (*P <* .05)

The findings showed that satisfaction scores for women served in public facilities (*M* = 89.61) were higher (*P <* 0.05) than satisfaction scores for women served in private (*M* = 83.39) or Marie Stopes (*M* = 82.73) clinics. Nevertheless, satisfaction scores were not statistically significant between private and Marie Stopes clinics. The mean satisfaction score was significantly (*P <* 0.05) higher for women whose procedures were MVA (*M* = 86.51) than MA (*M* = 83.53). Women whose gestational age was beyond 12 weeks (*M* = 89.50) rated higher satisfaction (*P <* 0.05) than those before 12 weeks of gestation (*M* = 84.51).

### Level of satisfaction on each factor

Each score of the five factors were transformed to the four point scale (one to four) for the sake of comparison. Factors have the following order of mean scores: privacy and confidentiality (mean 3.43, SD .48), followed by technical quality of care provider (mean 3.33, SD .58), art of care (mean 3.32, SD .49), information (mean 3.28, SD .50), and physical environment (mean 3.02, SD .70).

## Discussion

In Ethiopia where unsafe abortion accounts 32% of maternal deaths (3), a local and valid measure of women’s perception of the quality of abortion care service is non-existent. This study was made to fill this gap.

Patient satisfaction, though narrower in scope than women’s experience [15], seems to be more meaningful construct to evaluate quality of care from the perspectives of women in Addis Ababa, Ethiopia.

Although the country is increasing its budget and resources allocated to women health care services, maternal mortality remains a major public health concern (3). Dissatisfied women do not adhere to the advice of clinicians, they may not return for follow-up visits and ultimately compromise their health (5). Hence, a client-centered approach would be a concrete approach for health systems to evaluate women’s experiences to serve them better. This tactic would also assist health facilities to provide quality services, ensure higher client satisfaction and eventually increase institutional performance in improving community health outcomes.

The primary purpose of this study was identifying factors associated with women’s satisfaction with CAC. In the same way that patients satisfaction is multidimensional [15–17], this study has shown that women’s satisfaction with CAC has five main underlying factors: art of care, physical environment, information, privacy and confidentiality, and quality of care providers.

Following identification of factor structure, levels of satisfaction and its variability were investigated across groups of respondents. Women who took part in this study reported that they were generally highly satisfied with the care. This is consistent with the findings of the study conducted in Oromia and Amhara regional states of Ethiopia that the majority of women rated high satisfaction with abortion services [18–20]. The skewed distribution of satisfaction scores toward the upper tail indicates that the service delivery was reasonably good from the customers’ standpoint. A study conducted in the United States of America (USA) has revealed that women rated their abortion care experience very positively 9.4 on a scale of 10 [15]. Given that the USA and Ethiopia are at the extreme variance in terms of development, the high satisfaction scores in both countries seem to be one indicator of validity of the measure obtained in this study.

The fact that clients were more satisfied in public health facilities than private or Marie Stopes clinics could be related with cost of the care. Studies have shown higher patient satisfaction associated with lower health care expenditures [21, 22]. The cost incurred for CAC services is far lower in public health facilities. Patient satisfaction and expectation of health care services are found to be negatively correlated [16]. The reason for higher satisfaction using MVA could be related to expectation that MVA is more painful, unsafe and more risky. Higher satisfaction at later gestational ages could also be due to a perception that late abortions are more complicated and painful.

Unlike other studies [15, 23] the relationship between age and satisfaction was not statistically significant. Since the raw scores of age and satisfaction were found to be statistically related (*r* = .137, *p* = .006) using Pearson Product Moment Correlation, grouping scores of the age variable could cause the first finding to be insignificant. As Cohen [24] stated, dichotomizing one of the variables to be correlated is as much as losing 38% (and 60% loss if both variables are dichotomized) of the power of the test.

In this study art of care was identified to be one of the major factors explaining satisfaction with CAC. As previous studies showed [24, 25], such respectful treatments as provider’s patience, concern, and attentiveness have a crucial impact on patient satisfaction. Given the sensitive nature of abortion care, it is not surprising that this factor was the most important factor considered.

As in other studies [26], pleasantness of the physical environment around the treatment room was found to be of high importance to women receiving the care. This suggested that physical environment should be targeted in quality improvement efforts.

The perceived adequacy of information women received was found to be important factor associated with women’s overall satisfaction, indicating that provision of all the desired information is an essential part of high-quality abortion services. Information must be complete, accurate and easy to understand, and be given in a way that facilitates a woman being able to freely give her fully informed consent, and is sensitive to her needs and perspectives [27].

Services should be delivered in a way that guarantees women’s right to privacy. Lack of privacy may discourage clients particularly adolescents and unmarried women, from seeking safe and legal abortion services, and may drive them to undergo unsafe abortion. Privacy and confidentiality is a key principle of medical ethics and must be guaranteed [21]. Health care providers therefore should guarantee this to be the case to women during conversations as well as during actual services.

In spite of the fact that women may have an imperfect knowledge to appraise the technical skills of their providers [2], it is fascinating that their trust in the technical competence of their providers was associated with their overall satisfaction ratings. Since women may have experienced illegal abortions or may have heard stories of unsafe abortions, ensuring the technical competence and confidence of provider would be very essential in this context.

The strength of this study is that suggestions given by known instrument development studies [16, 28, 29] have successfully been integrated in developing items measuring women’s satisfaction with CAC services, suggesting that satisfaction measures are reliable and valid. This study also has limitations. Firstly, it was conducted within narrow scope of study area and population. Secondly, data were gathered at one specific point in time, so the study contains the typical limitations related with cross-sectional research designs. None response rates both in the qualitative and quantitative part of the study can also be considered as limitation of this study. However, since the missing participants varied somewhat uniformly in terms of key socio-demographic variables, loss of those participants might not have significant impact on the results of the study.

## Conclusion

Despite the aforementioned limitations, this research extremely useful for decision makers offering abortion care services in Ethiopia. In particular, this study improves the understanding of how women perceive abortion service quality. The findings suggest that abortion care satisfaction is a multidimensional construct explained by five major factors. Therefore, to improve the overall quality of CAC services, providers, managers and policy makers should give specific attention to the advancement of these components namely, positive client-provider interaction, pleasantness of physical environment, offering adequate information related to abortion procedures, securing clients’ privacy during counseling and treatment, and the technical quality of the providers.

## References

[1] World Health Organization (2011). Unsafe abortion: global and regional estimates of the incidence of unsafe abortion and associated mortality in 2008.

[2] World Health Organization (2012). Safe Abortion: Technical and Policy Guidance for Health Systems.

[3] Federal Ministry of Health of Ethiopia (2006). Technical and Procedural Guidelines for Safe Abortion Services in Ethiopia.

[4] Ethiopian Parliament. The Criminal Code of the Federal Democratic Republic of Ethiopia, 2004

[5] Engender Health, Ipas (2009). COPE® for comprehensive abortion care: a toolbook to accompany the COPE handbook. Engender Health Quality Improvement Series.

[6] Billings D, Fuentes Velásquez J, Pérez-Cuevas R (2003). Comparing the quality of three models of post abortion care in public hospitals in Mexico City. Int Fam Plan Perspect.

[7] Singh S, Fetters T, Gebreselassie H, Abdella A, Gebrehiwot Y, Kumbi S (2010). The estimated incidence of induced abortion in Ethiopia, 2008. Int Perspect Sex Reprod Health.

[8] Gebreselassie H, Fetters T, Singh S, Abdella A, Gebrehiwot Y, Tesfaye S (2010). Caring for women with abortion complications in Ethiopia: national estimates and future implications. Int Perspect Sex Reprod Health.

[9] Jewkes RK (2005). Why are women still aborting outside designated facilities in metropolitan South Africa?. BJOG.

[10] Kathryn AM, David AC, Susan MG (2004). The Role of Clinical and Process Quality in Achieving Patient Satisfaction in Hospitals. Decis Sci.

[11] Elaine Y, Gail CD, Richard R (2002). The Measurement of Patient Satisfaction. J Nurs Care Qual.

[12] Pett M, Lackey N, Sullivan J (2003). Making Sense of Factor Analysis: The use of factor analysis for instrument development in health care research.

[13] Tabachnick B, Fidell L (2001). Using Multivariate Statistics.

[14] Kline P (1994). An easy guide to factor analysis.

[15] Taylor D, Postlewaite D, Desai S, James EA, Calhoun AW, Sheehan K (2013). Multiple determinants of the abortion care experience: From the patient’s perspective. Am J Med Qual.

[16] Ware JE, Snyder MK, Wright R, Davies AR (1983). Defining and measuring patient satisfaction with medical care. Eval Program Plann.

[17] Risser N (1975). Development of an instrument to measure patient satisfaction with nurses and nursing care in primary care settings. Nurs Res.

[18] Solomon K, Yilma M, Hailu Y (2003). Quality of post-abortion care in public health facilities in Ethiopia.

[19] Hu D (2009). Cost-effectiveness analysis of alternative first-trimester pregnancy termination strategies in Mexico City. BJOG.

[20] Lewis JR (1994). Patient views on quality care in general practice: literature review. Soc Sci Med.

[21] Joshua JF, Anthony FJ, Klea DB, Peter F (2012). The Cost of Satisfaction: A National Study of Patient Satisfaction, Health Care Utilization, Expenditures, and Mortality.

[22] Marlene M, Elisabeth D, Margareta L (2012). Women and men’s satisfaction with care related to induced abortion.

[23] Cohen J (1983). The Cost of Dichotomization. Appl Psychol Meas.

[24] Crow R, Gage H, Hampson S (2002). The measurement of satisfaction with healthcare: implications for practice from a systematic review of the literature. Health Technol Assess.

[25] Donabedian A (2003). The Definitions of Quality and Approaches to Its Assessment.

[26] Wiebe ER, Sandhu S (2008). Access to abortion: what women want from abortion services. J Obstet Gynaecol.

[27] Sedgh G (2012). Induced abortion: incidence and trends worldwide from 1995 to 2008. Lancet.

[28] DeVellis RF (2012). Scale Development: Theory and applications.

[29] DeVellis RF (2003). Scale Development: Theory and applications.

